# Non-Traumatic Clavicular Lesions in Children: Case Series and Literature Review

**DOI:** 10.3390/children13010112

**Published:** 2026-01-12

**Authors:** Federico Diomeda, Rossella Greco, Paola Lazzari, Giulia Loiacono, Manuela Taurisano, Adina Pinna, Francesco La Torre, Alessandro Cocciolo, Luca Giordano, Flavia Indrio, Arnaldo Scardapane, Angelo Ravelli, Adele Civino

**Affiliations:** 1 Reumatologia e Immunologia Pediatrica, Ospedale ‘Vito Fazzi’, 73100 Lecce, Italy; federicodiomeda91@gmail.com (F.D.); gl.giulialoiacono@gmail.com (G.L.); adinap1991@gmail.com (A.P.); adelecivino@gmail.com (A.C.); 2 Dipartimento Interdisciplinare di Medicina, Università degli Studi di Bari ‘Aldo Moro’, 70121 Bari, Italy; greco.rossella1994@gmail.com (R.G.); lazzari.paola94@gmail.com (P.L.); 3 Dipartimento di Scienze e Tecnologie Biologiche ed Ambientali, Università del Salento, 73100 Lecce, Italy; 4 Centro di Reumatologia Pediatrica, Policlinico di Bari, 70124 Bari, Italy; reumapedlatorre@gmail.com; 5 Oncoematologia Pediatrica, Ospedale ‘Vito Fazzi’, 73100 Lecce, Italy; acocciolo@live.it; 6 Reparto di Radiologia dell’Ospedale ‘Vito Fazzi’, Università del Salento, 73100 Lecce, Italy; 76giordanol@gmail.com (L.G.); arnaldo.scardapane@unisalento.it (A.S.); 7 Dipartimento di Medicina Sperimentale, Sezione di Pediatria, Ospedale ‘Vito Fazzi’, Università del Salento, 73100 Lecce, Italy; flavia.indrio@unisalento.it; 8 Direzione Scientifica, IRCCS Istituto Giannina Gaslini, 16147 Genova, Italy; angeloravelli@gaslini.org; 9 Dipartimento di Neuroscienze, Riabilitazione, Oftalmologia, Genetica e Scienze Materno-Infantili (DINOGMI), Università degli Studi di Genova, 16126 Genova, Italy

**Keywords:** clavicle, non-traumatic clavicular lesions, chronic nonbacterial osteomyelitis (CNO), osteomyelitis, arthritis, bone lesions, whole-body MRI, pediatric rheumatology, clavicular pain, children

## Abstract

Background and Objective: Clavicular pain and swelling in children can have multiple causes and often require a multidisciplinary approach. We aimed to describe the characteristics and final diagnoses of children with clavicular involvement and to review the literature on this topic. Methods: We retrospectively reviewed patients younger than 18 years who were evaluated for clavicular symptoms at two pediatric rheumatology centers and one pediatric oncohematology center. These data were then descriptively compared with findings from 63 patients reported across 7 published articles. Results: Twelve patients (9 females, median age 10 years [IQR 9.4–10.5]) were included. Final diagnoses were chronic nonbacterial osteomyelitis (CNO; 8), Langerhans cell histiocytosis (LCH; 2), reactive arthritis (1), and Tietze syndrome (1). Clavicular involvement was mostly unilateral and localized to the medial clavicle in CNO. The most frequent presenting symptom was local swelling (11/12), followed by pain (9/12). Diagnostic delay was a median of 4 months (IQR 1–10.5). Whole-body MRI revealed multifocal lesions in 6/8 CNO patients. Biopsy was often required for diagnosis primarily to exclude malignancy and to clarify atypical or unifocal presentations. The literature review confirmed CNO as the most frequent cause, followed by rare tumors. Conclusions: CNO predominates among pediatric non-traumatic clavicular lesions, but LCH and rare conditions are not uncommon, underscoring the need for careful differential diagnosis and targeted imaging.

## 1. Introduction

The clavicle can be affected by a range of conditions—traumatic as well as non-traumatic—including infectious, oncologic, and inflammatory disorders. The appearance of symptoms such as clavicular pain and swelling in pediatric patients can raise significant concern, as these signs necessitate a thorough differential diagnosis and a comprehensive multidisciplinary assessment. Non-traumatic clavicular pathology in children is rare and infrequently documented in the literature, with an estimated incidence of 0.38 cases per 100,000 children per year [1]. Non-traumatic pathologies affecting this area can be classified as either tumor related—such as Ewing’s sarcoma, osteosarcoma, and Langerhans cell histiocytosis—or non-tumor related, including chronic nonbacterial osteomyelitis (CNO) and bacterial osteomyelitis [2,3,4].

Due to the non-specific nature of early symptoms, distinguishing between these pathologies can be challenging, often resulting in delayed diagnosis. In this context, collaboration among various professionals, such as radiologists, pediatric oncologists, pediatric rheumatologists, and pathologists, is crucial to ensure timely and appropriate diagnosis. The aim of this study is to present a series of patients in whom clavicular symptoms were the initial manifestations and to review the literature on this topic, offering an overview of the diagnostic methods employed and emphasizing the importance of an integrated approach to clinical management. Through the analysis of these experiences, the study aims to contribute to the understanding of the clinical and diagnostic implications of seemingly mild symptoms which, however, may be associated with aggressive pathologies.

## 2. Materials and Methods

We collected retrospective data on consecutively referred pediatric patients (≤18 years) referred to pediatric rheumatology and oncohematology centers in the Apulia region (Italy) between 2009 and 2024, presenting with non-traumatic clavicular involvement at onset. All cases required written informed consent.

The study was conducted in accordance with Italian regulations for non-pharmacological observational studies and the principles of the Declaration of Helsinki. Written informed consent was obtained from all participants or their legal guardians. Clinical, diagnostic and demographic data collected included: age at diagnosis, sex, site of clavicular involvement, local and systemic symptoms at onset, pain characteristics, involvement of other organs, presence of multifocal lesions, laboratory findings, imaging studies, histology, initial misdiagnoses, and time to diagnosis.

To gather additional information on the topic, we conducted a literature review to identify relevant articles concerning non-traumatic clavicular lesions in children and adolescents. The literature review was conducted following the PRISMA guidance in an informal manner, given the exploratory and descriptive nature of the review. A search was performed in PubMed for articles published in English, with no restrictions on publication year, up to 31 October 2025. The search string employed was: ((“clavicle”[Title/Abstract] OR “clavicular”[Title/Abstract] OR “clavear”[Title/Abstract]) AND (“pediatric”[Title/Abstract] OR “child”[Title/Abstract] OR “children”[Title/Abstract])) AND (“lesion”[Title/Abstract] OR “tumor”[Title/Abstract] or “cancer”[Title/Abstract] or “disorder” [Title/Abstract] or “involvement” [Title/Abstract). We included articles in this review if they comprised case series or case–control studies, aged 0–18 years with non-traumatic clavicular lesions. Study selection was performed by FD and independently reviewed by AC, whereas data extraction was conducted by PL.

Data collection and descriptive statistical analyses were performed using Microsoft Excel for Mac (version 16.99.2; Microsoft Corp., Redmond, WA, USA), including the calculation of means and frequency distributions.

## 3. Results

We included 12 patients with clavicular involvement as a presenting sign, with a median age at diagnosis of 10 years (range 9.4–10.5). Females were 9/12 (75%). Diagnosis is reported as follows: chronic non-bacterial osteomyelitis (CNO) (8/12–67%), Langerhans cell histiocytosis (LCH) (2/12–17%) Tietze syndrome (1/12–8%), and reactive arthritis (1/12–8%). Comorbidities included thyroiditis (1/12–8%), coeliac disease (2/12–17%), and juvenile idiopathic arthritis (1/12–8%).

In ten cases (83%), clavicular localization was unilateral (three in the left clavicle, five in the right clavicle, and two in the sternoclavicular joint) and in other two cases (17%), it was bilateral (one in both clavicles and one in the sternoclavicular joint).

Medial clavicle involvement was the most common localization (7/12–58%). Swelling as an onset symptom was reported in 11/12 (91%) patients, in most cases associated with pain. Morning stiffness did not affect any of the patients. In two cases of CNO, pain was reported worsening at night and only four patients (4/11–36%) reported relief with non-steroidal anti-inflammatory drugs (NSAIDs) (two CNO, one reactive arthritis, one Tietze’s syndrome).

Regarding extraskeletal manifestations, only patients with a diagnosis of LCH showed widespread lymphadenomegaly. In one case, skin involvement was observed at symptom onset.

Considering the entire cohort, the median time between the onset of symptoms and diagnosis was 4 months (IQR range 1–10.5). These data are summarized in Table 1.

Laboratory tests were available for 11 patients, with elevated erythrocyte sedimentation rate (ESR) and/or C-reactive protein (CRP) recognizable in six cases (five CNO and one LCH); lactate dehydrogenase (LDH) was elevated only in LCH cases.

Ultrasonography was performed in three patients (one reactive arthritis, one Tietze syndrome, and one CNO). In the patient with reactive arthritis, ultrasound revealed a sternoclavicular joint effusion. Plain radiographs were available for eleven patients, with pathological bone findings in five patients: three osteolysis and two uneven bone density.

Eight patients underwent whole body magnetic resonance imaging (MRI), identifying seven patients having multifocal distribution of musculoskeletal lesions (six with CNO, one with LCH). The most frequent extraclavicular locations were femur metaphysis (3/7), tarsus (3/7), tibial metaphysis (3/7). WBMRI was performed at diagnosis as a second level imaging method.

**Figure 1:**
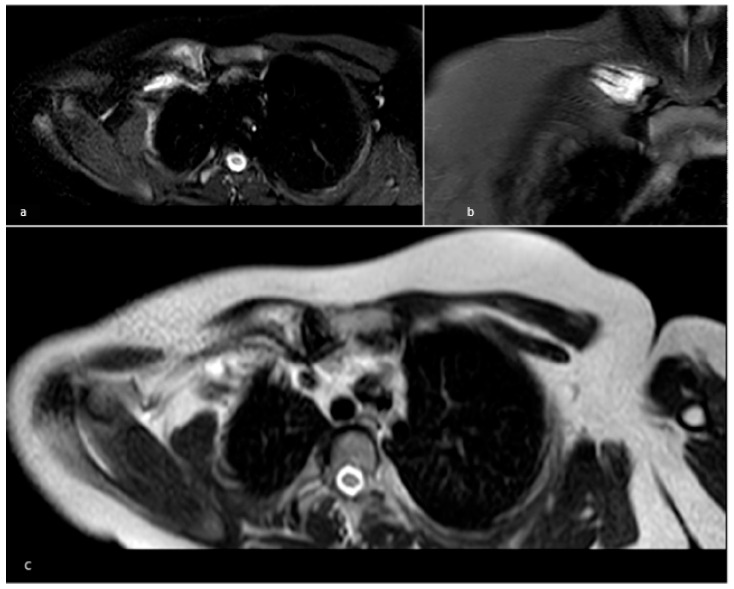
Patient 6, diagnosed with LCH. (**a**) Whole-body MRI showing a focal area of hyperintensity in the long TR sequences (STIR image) at the medial third of the right clavicle with cortical swelling and periosteal reaction; (**b**) Coronal STIR image clearly showing the hyperintensity area in STIR; (**c**) T2-weighted sequence highlighting both the morphological and signal alteration (hyperintensity) of the medial third of the right clavicle.

**Figure 2:**
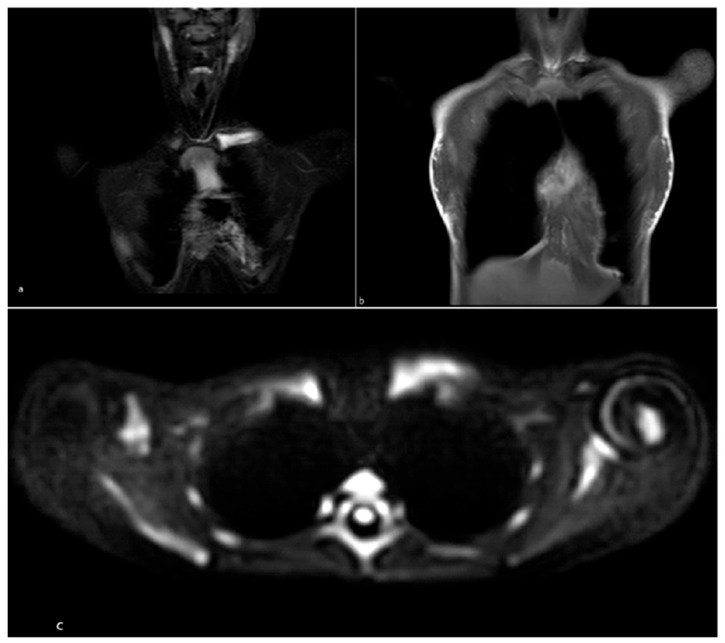
Patient 3 with diagnosis of CNO. (**a**) Whole-body MRI, T2 STIR coronal: marked signal hyperintensity at the medial end of the left clavicle; (**b**) Coronal T1 image showing hypointensity at the same site; (**c**) DWIbs (Diffusion Weighted Imaging with background suppression).

**Figure 3:**
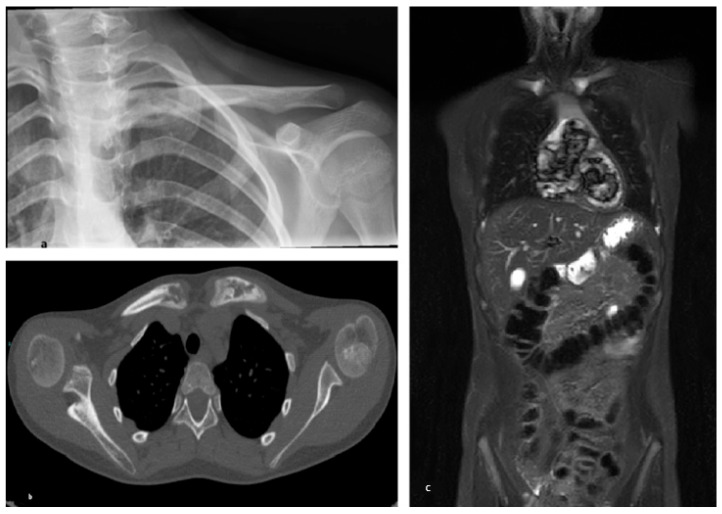
Patient 1, diagnosed with JIA in overlap with CNO. (**a**) X-ray of the left clavicle showing focal osteolytic changes at the medial end of the left clavicle; (**b**) CT scan showing osteolytic changes at the sternal ends of both clavicles; (**c**) Whole-body MRI (STIR) showing focal hyperintensity at both medial clavicular ends.

Figure 1, Figure 2 and Figure 3 illustrate in detail the MRI features of three representative patients with CNO.

Biopsy of the clavicular lesion was performed in eight patients, in cases with solitary lesions, atypical imaging findings, or when malignancy could not be confidently excluded. Histopathological examination confirmed a diagnosis of CNO in all of these cases. Patients with a diagnosis of LCH obtained a confirmation of diagnosis undergoing lymph node biopsy.

The search terms yielded 146 references. From this group, we excluded single case reports, traumatic clavicular lesions, studies in which the clavicle was not the primary site investigated, and those including mixed adult cohorts, resulting in a final selection of seven references.

Given the limited number of relevant publications and the narrative scope of the review, all identified articles were included and discussed qualitatively. Therefore, no formal screening numbers or quality assessment were performed.

Articles founded collectively encompassed 63 patients (30 female and 33 male). The median age at diagnosis was 11.3 years. Articles were published across multiple disciplines: one clinical medicine journal, one general pediatrics journal, one pediatric oncohematology journal, two orthopedics journals, one pediatric orthopedics journal, and one pediatric radiology journal.

Among the 63 pediatric cases identified, the most common diagnoses were CNO/chronic osteomyelitis (10 cases), condensing osteitis (14 cases), Ewing sarcoma (9 cases), and eosinophilic granuloma (7 cases). When reported, presenting symptoms included localized pain and swelling in virtually all the subjects. Systemic manifestations—such as fever or cutaneous involvement—were not consistently available across all studies. A biopsy of the clavicular lesion was performed in most patients, allowing histopathological confirmation of the diagnosis. Data extracted from the literature are summarized in Table 2.

## 4. Discussion

Although non-traumatic clavicular involvement in children is rare, accurate characterization of its clinical and radiological features is essential because of the broad range of potential differential diagnoses. Failure to recognize the underlying cause may result in diagnostic and therapeutic delays, especially in cases with potentially poor outcomes, such as malignant lesions.

Data from our cohort, from other similar case series, and from larger analyses consistently show that the most frequent cause of pain and/or swelling of the clavicle is osteomyelitis [1,8,9].

The term *osteomyelitis* refers to bone inflammation, which may be of either infectious or aseptic origin. Although infectious osteomyelitis can be recognized, most pediatric cases are expressions of CNO. Moreover, even in cases presumed to be of infectious origin, biopsies frequently failed to identify any causative microorganism [10,11]. In a small proportion of cases, culture negativity may reflect *Mycobacterium tuberculosis* infection, since isolation of the pathogen is often difficult [12].

A problem of terminology exists regarding CNO, especially in earlier reports in which it was referred to as *chronic osteomyelitis* or *condensing osteitis* of the clavicle [1,6,7]. CNO is a relatively recent term that has replaced the historical designation of CRMO, reflecting advances in disease understanding, whereas the term condensing osteitis is much older and derives from earlier more descriptive radiological classifications.

From the analysis of the literature, it emerges that reports of clavicular involvement in CNO have declined over time, reflecting an improved recognition of the disease as a systemic rather than localized process. In the new EULAR/ACR classification criteria for CNO in children, the presence of an inflammatory lesion on imaging located in the clavicle alone contributes 17 out of 55 points to the total score [13].

CNO is an autoinflammatory disorder characterized by recurrent or episodic sterile bone inflammation. It primarily affects children between 7 and 12 years of age, though cases in adults have also been reported. The clinical presentation is variable, ranging from asymptomatic or mild monofocal lesions to severe localized pain involving multiple skeletal sites [13,14].

Our cohort, similarly to what has been reported in the literature, was mainly composed of patients with CNO. This predominance influenced the overall demographic profile, which showed a female majority (75%) and a mean age at diagnosis of 10 years.

Clement et al. collected pediatric cases of medial clavicular tumors from multiple European registries and concluded that malignancies in this location are exceptionally uncommon (one case every 275 child-years at risk), with most lesions representing benign or inflammatory conditions [11]. After the exclusion of potential mimickers, clavicular involvement in older children can therefore be considered virtually pathognomonic for CNO [15]. This concept becomes less relevant in adults, where the clavicle is more often involved by malignant lesions, displaying a gradient of risk that rises with advancing age [16].

Neoplasms of the clavicle are rare, representing less than 1% of all primary bone tumors [17]. In a series of 27 clavicular lesions from the Bone Tumor Database of a tertiary referral Orthopedic Oncology Center, no malignant cases were identified. Five lesions were benign—mostly cystic in nature—while the majority were consistent with CNO [18]. In our cohort, we observed two cases of LCH involving the clavicle. *Eosinophilic granuloma*, the term used for single system and single site involvement of LCH, appears to be the second most frequent disease encountered and the most common tumor [1,2,19]. In pediatric cases, LCH most often presents with isolated bone involvement, occurring in approximately 73% of patients. Multifocal skeletal disease is seen in about 25% [20]. Clavicular localization is rare, reported in only 3.1%, occurring predominantly in the medial portion, although with a lower medial/lateral ratio compared to CNO [2,10,21]. Among the six CNO patients and the one case of multifocal LCH, the most frequently affected extraclavicular sites were femoral metaphysis, tarsal bones, and tibial metaphysis.

Studies on LCH have shown that it typically presents at a younger age than CNO, with a median onset between 3 and 4 years of age [19]. This age difference was also reflected in our cohort: the youngest patient, diagnosed at 8 months of age, had LCH. These age-related patterns are clinically relevant, as they can help guide the diagnostic process. In our case series, no malignant tumors were observed. However, in the literature, studies reporting malignant clavicular lesions frequently describe cases of Ewing’s sarcoma in children [Table 2] [10]. Ewing’s sarcoma is an aggressive tumor that primarily involves the bones and, less commonly, the soft tissues. It predominantly affects children and adolescents, with a median age at diagnosis of approximately 15 years. While Ewing’s sarcoma can arise in any bone, in pediatric populations, it most commonly affects flat bones, such as the pelvis and chest wall, and long bones of the limbs [22]. Clavicular involvement is rare, accounting for approximately 1.4% of all cases, and no consistent pattern of localization (lateral, medial, or middle) has been reported in this condition [9] When evaluating a child with musculoskeletal involvement in a pediatric rheumatology or oncohematology setting, the possibility of an underlying neoplasm is often considered. This aspect has been explored, and a score has been proposed to support the differentiation of these conditions at onset. This score was primarily developed for the most common pediatric malignancies, such as acute lymphoblastic leukemia, and may assist in assessing the likelihood of inflammatory arthropathy versus musculoskeletal involvement secondary to leukemia [23,24].

Local symptoms involving the clavicle varied among patients. Pain and swelling were the most frequently reported manifestations, both in our cohort and in the literature [Table 2]. Because these manifestations, particularly pain, may occur in otherwise healthy children, they can initially be overlooked or mistaken for benign conditions [25]. In our study, two patients with CNO were initially misdiagnosed as having clavicular subluxations, but these misclassifications did not lead to a prolonged diagnostic delay. In our cohort, the median diagnostic delay was 4 months, which is significantly shorter than that reported in the literature for patients with CNO [4]. Although the number of patients was limited and a formal quantitative analysis could not be performed, a generally longer diagnostic delay could be observed in our patients with CNO compared with those with LCH.

CNO patients in our cohort did not report any extra osseous manifestations, while both LCH patients presented with generalized lymphadenopathy.

From a laboratory perspective, elevated ESR and/or CRP were observed in most patients with CNO in our cohort (62%), whereas increased LDH was detected only in the two patients with LCH. LDH may be elevated in neoplastic conditions due to increased cellular turnover and may serve as a supportive screening marker [26]. For this reason, it is considered an exclusion criteria for CNO diagnosis in the recent EULAR/ACR criteria [13].

Plain radiography is the first-line imaging modality for the evaluation of clavicular disorders; standard radiographs in two projections should be obtained, including the sternoclavicular and/or acromioclavicular joints. In the presence of persistent pain, swelling, fever, or elevated inflammatory markers, a normal X-ray does not exclude significant pathology, and further imaging is required.

In our cohort, the majority of cases are presented with normal radiographs. This aligns with findings from CNO studies, which have reported normal radiographs in up to 80% of cases, particularly in the early stages of the disease [27].

MRI plays a central role in patients presenting with bone pain with or without fever and in cases of suspected osteomyelitis or sternoclavicular arthritis. It provides a comprehensive evaluation of bone marrow, cortical integrity, soft-tissue extension, collections, and joint involvement, and is therefore essential for defining disease extent.

Our data confirm that WB MRI is the best imaging option for CNO, as it avoids ionizing radiation and is highly sensitive in detecting multifocal bone involvement and early inflammatory changes, such as bone marrow edema. MRI is also considered the most appropriate method for long-term monitoring due to its high sensitivity [13,14]. CT is not routinely indicated in this setting but may be useful for detailed analysis of lesion matrix or cortical architecture and for surgical planning.

Biopsy was performed in 10 out of 12 patients in our cohort: specifically, two lymph node biopsies in patients with LCH and eight biopsies of clavicular lesions in patients with CNO. Although the current literature generally recommends biopsy for solitary or atypical multifocal lesions in CNO, histopathological analysis remains essential to rule out malignancies. With increasing awareness and improved diagnostic criteria, clinicians have progressively reduced the use of biopsy in the diagnostic workup of CNO [14,15]. The limitations of our study include its retrospective design, small sample size, and the absence of detailed data on therapeutic approaches. In addition, as patients were referred to tertiary pediatric rheumatology and oncohematology centers, a referral-center bias cannot be excluded. Furthermore, due to the scarcity of similar studies in the literature, comparisons were primarily made with cohorts focused on CNO. Nevertheless, the strength of our study lies in its novelty, as it addresses rare and often underreported conditions involving the pediatric clavicle.

In conclusion, non-traumatic clavicular lesions are rare in pediatric patients and may present significant diagnostic challenges at initial evaluation. While CNO emerged as the most frequent diagnosis in both our cohort and the literature, clinicians should remain vigilant for alternative etiologies, including malignant lesions.

## Figures and Tables

**Table 1:** Demographic and clinical characteristics of the study cohort.

Patient No.	Sex	Diagnosis	Age at Diagnosis (Years)	Time to Diagnosis (Months)	Location	Local Symptoms at Onset	Other Symptoms at Onset	Clinical Response to NSAIDs
1	F	CNO	9.5	1	Bilateral clavicle, medial	Pain, swelling	No	Yes
2	F	LCH	0.6	0	Bilateral sternoclavicular joints	Swelling	Lymphadenopathy, skin lesions	No
3	F	CNO	10.3	2	Left clavicle, lateral	Pain, swelling	No	Yes
4	F	Reactive arthritis	10.3	4	Right sternoclavicular joint	Pain, swelling	No	Yes
5	F	Tietze syndrome	15.4	1	Left sternoclavicular joint	Pain	No	Yes
6	F	LCH	10.8	1	Right clavicle, medial	Pain, swelling	Lymphadenopathy	No
7	F	CNO	10.0	12	Right clavicle, medial	Pain, swelling	Fever, inappetence	NA
8	F	CNO	7.0	5	Right clavicle, medial	Pain, swelling	No	No
9	F	CNO	9.9	10	Right clavicle, lateral	Pain, swelling, warmth on palpation	No	No
10	M	CNO	10.0	15	Left clavicle, medial	Swelling	No	No
11	M	CNO	12.6	23	Bilateral clavicles, medial	Pain, swelling	No	No
12	M	CNO	9.1	4	Right clavicle, medial	Pain, swelling	No	No

**Table 2:** Analysis of the current literature regarding clavicular pediatric non-traumatic lesions.

Study, Year	No. of Patients	Mean Age at Diagnosis (Years)	Gender	Location	Symptoms	Diagnosis	Biopsy
JIANG et al., 2024 [2]	20	10	7/20 F	11/20 medial 7/20 lateral 2/20 middle	13 swelling 6 swelling and pain 1 no symptoms	5 eosinophilic granuloma 3 enchondromatosis 3 osteochondroma 2 chronic osteomyelitis 2 bone cyst 1 bone tuberculosis 1 acute osteomyelitis 1 hemangioendothelioma 1 chondromyxoid fibroma	20/20
RADHAKRISHNAN et al., 2010 [3]	4	16	4/4 F	N/A	4 pain and swelling	4 Ewing sarcoma	4/4
RODRIGUEZ MARTIN et al., 2009 [5]	5	11.6	2/5 F	1/5 medial 2/5 lateral 2/5 middle	5 pain and swelling	5 Ewing sarcoma	5/5
ANDREACCHIO et al., 2016 [6]	7	11.5	2/7 F	7/7 medial	7/7 swelling 5/7 pain and swelling	7 Condensing osteitis of the clavicle	5/7
APPEL et al., 1983 [7]	7	11.1	7/7 F	7/7 medial	7/7 pain and swelling	7 Condensing osteitis of the clavicle	7/7
FRANKLIN et al., 1987 [1]	16	7.3	5/16 F	N/A	5/16 swelling, 2/16 pain, 9 pain and swelling	1 Fibrous dysplasia 2 Eosinophilic granuloma 1 Multipotential sarcoma 1 Osteochondroma 2 Aneurysmal bone cyst 4 Chronic osteomyelitis 2 Osteomyelitis 3 Congenital pseudarthrosis	16/16
DOCQUIER et al., 2006 [8]	4	12	3/4 F	4/4 medial	3/4 pain and swelling, 1/4 swelling	4 SAPHO/CRMO	3/4

## Data Availability

No new data were created or analyzed in this study. Data sharing is not applicable to this article.

## Abbreviations

The following abbreviations are used in this manuscript:

IQR	Interquartile Range
CNO	Chronic nonbacterial osteomyelitis
LCH	Langerhans cell histiocytosis
MRI	Magnetic Resonance Imaging
ESR	Erythrocyte sedimentation rate
CRP	C-reactive protein
LDH	Lactate dehydrogenase
STIR	Short Tau Inversion Recovery
DWI	Diffusion Weighted Imaging
JIA	Juvenile Idiopathic Arthritis
SAPHO	Synovitis, Acne, Pustulosis, Hyperostosis, Osteitis
CRMO	Chronic recurrent multifocal osteomyelitis
EULAR	European Alliance of Associations for Rheumatology
WBMRI	Whole-Body Magnetic Resonance Imaging

## References

[1] Franklin J.L., Parker J.C., King H.A. (1987). Nontraumatic clavicle lesions in children. J. Pediatr. Orthop..

[2] Jiang B., Li Q., Guo W., Ju L. (2024). Clinical Characteristics of Pediatric Clavicular Lesions: A Retrospective Analysis of 20 Cases. Cureus.

[3] Radhakrishnan V., Rastogi S., Bakhshi S. (2011). Ewing sarcoma of the clavicle: A case series. Indian Pediatr..

[4] Girschick H., Finetti M., Orlando F., Schalm S., Insalaco A., Ganser G., Nielsen S., Herlin T., Koné-Paut I., Martino S. (2018). The multifaceted presentation of chronic recurrent multifocal osteomyelitis. Rheumatology.

[5] Martin J.R., Mazzini J.P., Fernandez R.V., Ciruelos R.M., de la Mano A.C. (2009). Ewing sarcoma of clavicle in children: Report of 5 cases. J. Pediatr. Hematol. Oncol..

[6] Andreacchio A., Marengo L., Canavese F. (2016). Condensing osteitis of the clavicle in children. World J. Orthop..

[7] Appell R.G., Oppermann H.C., Becker W., Kratzat R., Brandeis W.E., Willich E. (1983). Condensing osteitis of the clavicle in childhood: A rare sclerotic bone lesion. Review of literature and report of seven patients. Pediatr. Radiol..

[8] Docquier P.L., Malghem J., Mousny M., Rombouts J.J. (2006). Chronic osteomyelitis of clavicle as primary manifestation of SAPHO syndrome in adolescents: Report of four cases and long-term evaluation. Jt. Bone Spine.

[9] Priemel M.H., Stiel N., Zustin J., Luebke A.M., Schlickewei C., Spiro A.S. (2019). Bone tumours of the clavicle. J. Bone Oncol..

[10] Hussain S., Khan Z., Akhtar N., Jeys L., Parry M., Grimer R.J. (2023). Anatomical distribution, the incidence of malignancy and diagnostic workup in the pathological lesions of the clavicle. Arch. Orthop. Trauma Surg..

[11] Clement N.D., Nyadu Y., Kelly M., Walmsley P., Porter D.E. (2011). Malignant lesions are rare in medial third of the clavicle in children: The European Juvenile Medial End of Clavicle Tumour study. J. Pediatr. Orthop. B.

[12] Hu W.R., Yao Z.L., Yu B., Jiang N. (2019). Clinical characteristics and treatment of clavicular osteomyelitis: A systematic review with pooled analysis of 294 reported cases. J. Shoulder Elb. Surg..

[13] Zhao Y., Oliver M.S., Schnabel A., Wu E.Y., Wang Z., Marino A., Aguiar C.L., Akikusa J.D., Akca U.K., Almeida B. (2025). EULAR/ACR classification criteria for pediatric chronic nonbacterial osteomyelitis (CNO). Ann. Rheum. Dis..

[14] Triaille C., De Bruycker J.J., Miron M.C., Lecouvet F., Girschick H., Wouters C. (2024). Update on the diagnosis and treatment of CNO in children: A clinician’s perspective. Eur. J. Pediatr..

[15] Roderick M.R., Shah R., Rogers V., Finn A., Ramanan A.V. (2016). Chronic recurrent multifocal osteomyelitis (CRMO)—Advancing the diagnosis. Pediatr. Rheumatol. Online J..

[16] Ren K., Wu S., Shi X., Zhao J., Liu X. (2012). Primary clavicle tumors and tumorous lesions: A review of 206 cases in East Asia. Arch. Orthop. Trauma Surg..

[17] Dahlin D.C., Unni K.K. (1996). Bone Tumors: General Aspects and Data on 8542 Cases.

[18] Suresh S., Saifuddin A. (2008). Unveiling the ‘unique bone’: A study of the distribution of focal clavicular lesions. Skelet. Radiol..

[19] Plasschaert F., Craig C., Bell R., Cole W.G., Wunder J.S., Alman B.A. (2002). Eosinophilic granuloma. A different behaviour in children than in adults. J. Bone Jt. Surg..

[20] Wang J., Wu X., Xi Z.J. (2010). Langerhans cell histiocytosis of bone in children: A clinicopathologic study of 108 cases. World J. Pediatr..

[21] Abdelaal A.H., Sedky M., Gohar S., Zaki I., Salama A., Hassanain O., El Ghoneimy A.M. (2020). Skeletal involvement in children with Langerhans cell histiocytosis: Healing, complications, and functional outcome. SICOT J..

[22] Grünewald T.G.P., Cidre-Aranaz F., Surdez D., Tomazou E.M., de Alava E., Kovar H., Sorensen P.H., Delattre O., Dirksen U. (2018). Ewing sarcoma. Nat. Rev. Dis. Primers.

[23] Civino A., Alighieri G., Prete E., Caroleo A.M., Magni-Manzoni S., Vinti L., Romano M., Santoro N., Filocamo G., Belotti T. (2021). Musculoskeletal manifestations of childhood cancer and differential diagnosis with juvenile idiopathic arthritis (ONCOREUM): A multicentre, cross-sectional study. Lancet Rheumatol..

[24] Civino A., Bovis F., Ponzano M., Alighieri G., Prete E., Sorrentino S., Magni-Manzoni S., Vinti L., Romano M., Santoro N. (2023). Development and Initial Validation of the ONCOREUM Score to Differentiate Childhood Cancer with Arthropathy from Juvenile Idiopathic Arthritis. J. Pediatr..

[25] Saffarzadeh M., Haydar S., Chan D., Andrews G., Ouellette H., Mallinson P., Munk P., Sheikh A. (2024). A clinico-radiological review of chronic non-bacterial osteomyelitis in paediatrics, adolescents, and adults: Demystifying a forgotten differential. Clin. Radiol..

[26] Jurisic V., Radenkovic S., Konjevic G. (2015). The Actual Role of LDH as Tumor Marker, Biochemical and Clinical Aspects. Adv. Exp. Med. Biol..

[27] Schaal M.C., Gendler L., Ammann B., Eberhardt N., Janda A., Morbach H., Darge K., Girschick H., Beer M. (2021). Imaging in non-bacterial osteomyelitis in children and adolescents: Diagnosis, differential diagnosis and follow-up—An educational review based on a literature survey and own clinical experiences. Insights Imaging.

